# A Metamaterials-Based Absorber Used for Switch Applications with Dynamically Variable Bandwidth in Terahertz Regime

**DOI:** 10.3390/ma17143571

**Published:** 2024-07-19

**Authors:** Yan Liu, Lingxi Hu

**Affiliations:** 1 School of Microelectronics, Shenzhen Institute of Information Technology, Shenzhen 518172, China; 2021000310@sziit.edu.cn; 2 Digital and Intelligent Agriculture Research Institute, School of Information Engineering, Huzhou University, Huzhou 313000, China

**Keywords:** terahertz absorber, terahertz switch, variable bandwidth, excellent absorptance

## Abstract

A broadband absorber based on metamaterials of graphene and vanadium dioxide (VO_2_) is proposed and investigated in the terahertz (THz) regime, which can be used for switch applications with a dynamically variable bandwidth by electrically and thermally controlling the Fermi energy level of graphene and the conductivity of VO_2_, respectively. The proposed absorber turns ‘on’ from 1.5 to 5.4 THz, with the modulation depth reaching 97.1% and the absorptance exceeding 90% when the Fermi energy levels of graphene are set as 0.7 eV, and VO_2_ is in the metallic phase. On the contrary, the absorptance is close to zero and the absorber turns ‘off’ with the Fermi energy level setting at 0 eV and VO_2_ in the insulating phase. Furthermore, other four broadband absorption modes can be achieved utilizing the active materials graphene and VO_2_. The proposed terahertz absorber may benefit the areas of broadband switch, cloaking objects, THz communications and other applications.

## 1. Introduction

Metamaterials, which usually consists of periodically arranged artificial unit cells with a sub-wavelength structure, possess outstanding electromagnetic properties [1]. Metamaterials-based applications have been found in many researching fields, such as metalenses [2], optical cloaking [3,4], sensors [5,6], biomedical research [7], and filters [8,9]. Since the first metamaterial absorber (MA) designed by Landy et al. in 2008 [10], a variety of MAs have been widely proposed and investigated, such as the absorbers with narrowband [11,12], multi-band [13,14,15], and broadband [16,17,18]. Among this research, metamaterials-based terahertz (THz) absorbers, working from 0.1 to 10 THz, have been given extensive attention for their promising application in future wireless communication [19,20]. However, the traditional MAs generally have only a single function, and the metamaterial layers are usually composed of periodically arranged metallic patterns, which are limited in practical applications for the fixed geometrical dimensions.

The reconfigurable characteristics of MAs can be realized by utilizing active materials such as phase transition materials [21,22], liquid metals [23], liquid crystals [24], ferroelectrics [25], photoconductive silicon [26,27], and 2D materials [28]. In recent years, graphene, with its unique electrical properties [29] such as high electron mobility, flexible tunability, relatively low loss and tight field confinement, has shown promising application in multifunctional THz devices [30,31,32,33,34]. The electromagnetic properties of the graphene-based MAs can be dynamically tuned by adjusting the Fermi energy level through chemical doping or electrostatic gating [35]. Moreover, vanadium dioxide (VO_2_), undergoing an insulator-metal transition process at the temperature around 340 K [36], plays a very active role in the switchable devices [37,38].

Zhang et al. have designed a MA based on strontium titanate and bulk Dirac semimetal. The absorption could be modulated in two modes, including single-band and dual-band, by thermally controlling the conductivity of VO_2_ and electrically changing the Fermi energy level of graphene [39]. Similar MAs based on active materials [40,41,42,43,44,45] usually work in one or two fixed frequency bands once the structure is fabricated, as shown in Table 1. Moreover, most MAs with two absorption modes show narrow relative bandwidth (RB) defined by RB = bandwidth/center frequency. Although a few absorbers show a relative large RB, the absorption broadband is fixed [44].

Herein, we proposed a metamaterials-based absorber in THz regime, which could be used for switch applications with multiple dynamic broadband absorption modes and large RB. When VO_2_ is in the metallic phase, and the Fermi energy levels of the graphene are simultaneously set as 0.7 eV, the broadband absorption can be achieved from 1.5 to 5.4 THz with the absorptance exceeding 90%, which indicates the state ‘on’ for switch application, and the relative bandwidth can reach 113%. When the Fermi energy level of graphene is 0 eV, the absorption of the proposed absorber exhibited a state transition from ‘on’ to ‘off’ by thermally controlling the conductivity of VO_2_ patterns and film. The physical mechanism of the broadband absorption is investigated by analyzing the distributions of the electric field and surface currents. Furthermore, the influence of the parameters on the broadband absorption is also investigated to guide the real fabrication of the proposed absorber. In addition, the other four broadband absorption modes can be realized by electrically and thermally adjusting the active material graphene and VO_2_, respectively. Generally, the proposed absorber promises switch application with various bandwidths.

## 2. Materials and Methods

**Figure 1:**
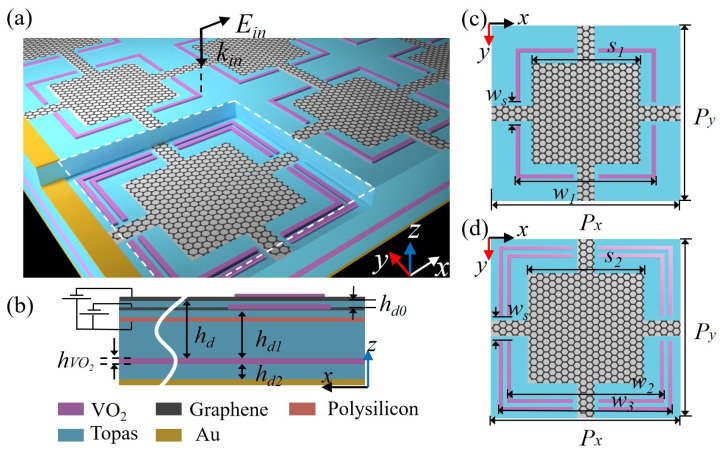
(**a**) Structure diagram of the broadband absorber with the polarization configuration of incident light. (**b**) Side view of the broadband absorber. (**c**) Top view of the unit cell for the upper, and (**d**) the lower metamaterial layer, respectively.

The proposed absorber consists of two metamaterial layers, a polysilicon sheet, a VO_2_ film, and a bottom gold (Au) plane, top to bottom, separating with polyethylene cyclic olefin copolymer (Topas) as depicted in Figure 1a,b. The connected graphene square and a VO_2_ split loop constitute the unit cell of the upper metamaterial layer, as shown in Figure 1c, and the unit cell of the lower metamaterial layer is also formed by the graphene square, apart from two VO_2_ split loops, as shown in Figure 1d. Both the width of the VO_2_ split loops as well as the spacer between the two VO_2_ split loops is fixed as 1 μm. The split gaps of the 200-nm-thick VO_2_ loops are set as *w*_s_. Topas is used as the insulating spacer for the proposed absorber, which has negligible loss and dispersion in the THz regime, and the relative permittivity of Topas is set as 2.35 [46]. The polysilicon sheet with extremely excellent semiconductor characteristics serves as the bottom electrode for controlling the Fermi energy level of graphene. The extremely thin (20 nm) polysilicon sheet has almost no influence on the absorption. The periods of the unit cell are *P*_x_ on the *x*-direction and *P*_y_ on the *y*-direction. The detailed dimensions of the proposed absorber are listed in Table 2.

The proposed absorber is numerically investigated by the finite element solver COMSOL Multiphysics (6.0, Stockholm, Sweden). The insulating phase and the metallic phase of VO_2_ are described with conductivities of 0 S/m and 2 × 10^5^ S/m, respectively. The optical characteristics of vanadium dioxide is defined by the dielectric permittivity, which is described by the Drude model [33]:

(1) $$\varepsilon \left(\omega \right)=\varepsilon_{\infty }-\frac{\omega_{p}^{2}\left(\sigma \right)}{\omega^{2}+i\gamma \omega },$$

where *ε*_∞_ = 12 is the dielectric permittivity at the infinite frequency, *γ* = 5.75 × 10^13^ rad/s is the collision frequency, and *ω*_p_(σ) is the plasma frequency. When *σ*_0_ = 3 × 10^5^ S/m, the value of plasma frequency is 1.4 × 10^15^ rad/s.

Graphene is simulated as a 2D material in the simulation. More specifically, graphene is modeled as a surface current *J* = *σ*_g_*E*_t_ in the frequency domain according to Ohm’s law, where *σ*_g_ is the conductivity of graphene and *E*_t_ is the tangential electric field on the graphene layer [47]. The complex surface conductivity of graphene can be described by the Kubo formula: *σ*_g_(*ω*, *τ*, *μ*_c_) = *σ*_intra_(*ω*, *τ*, *μ*_c_) + *σ*_inter_(*ω*, *τ*, *μ*_c_). The interband and intraband transition contributions are expressed as [48]:

(2) $$\sigma_{\mathrm{inter}}\left(w,\tau ,\mu_{c}\right)\approx \frac{je^{2}}{4\pi \hbar }\ln\left[\frac{2\left|\mu_{c}\right|-\left(\omega +j/\pi \right)\hbar }{2\left|\mu_{c}\right|+\left(\omega +j/\pi \right)\hbar }\right],$$

(3) $$\sigma_{\mathrm{intra}}\left(w,\tau ,\mu_{c}\right)\approx j\frac{e^{2}k_{B}T}{\pi \hbar^{2}\left(\omega^{2}+j\tau^{-1}\right)}\left[\frac{\mu_{c}}{k_{B}T}+2\ln\left(\exp\left(-\frac{\mu_{c}}{k_{B}T}\right)+1\right)\right],$$

where *T*, *ω*, *τ* = *μE*_f_*e*^−1^*υ*_F_^−2^, *k*_B_, *μ*_c_, and *ħ* ≈ 1.055 × 10^−34^ J·s are the temperature in Kelvin, incident angular frequency, relaxation time, Boltzmann constant, chemical potential, and reduced Planck constant, respectively. The chemical potential *μ*_c_ is equal to the Fermi energy level *E*_f_ for *k*_B_ < *μ*_c_. The relaxation time *τ* is 0.1 ps for *μ* = 1500 cm^2^V^−1^s^−1^ and *E*_f_ = 0.7 eV.

**Figure 2:**
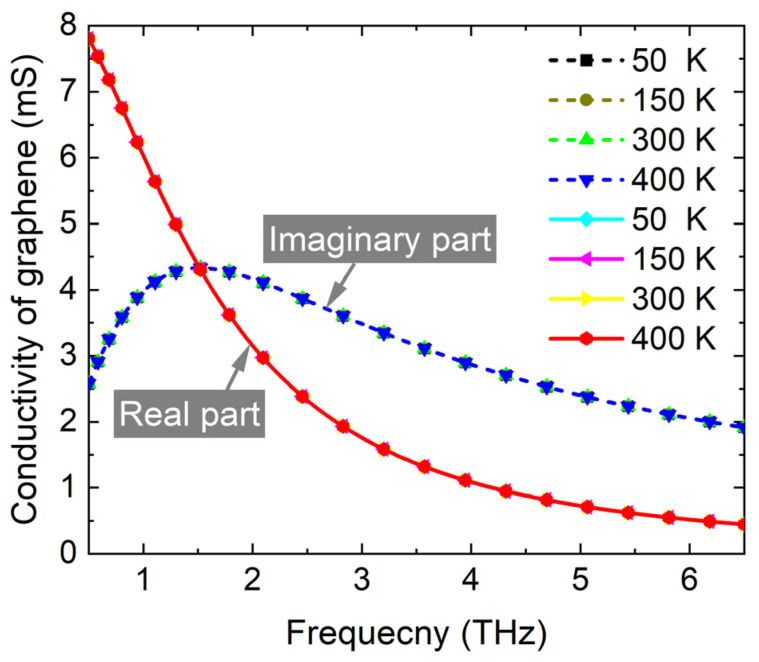
The conductivities of graphene vary with temperature from 0.5 to 6.5 THz. The carrier mobility *μ* is set as 1500 cm^2^V^−1^s^−1^ in this calculation. The solid curves illustrate the real part of conductivity, and the dash curves illustrate the imaginary part of conductivity.

According to the formula, the conductivity of graphene is a function related to the temperature. When the temperature varies from 50 K to 400 K, corresponding to the VO_2_ varying from metallic phase to insulating phase, the conductivity of graphene changes little in the frequency range from 0.5 to 6.5 THz, as shown in Figure 2. It is well known that the temperature of the surrounding environment is much lower than 340 K, at which the VO_2_ undergoes an insulator–metal transition process. The bias voltage applied on the graphene layer has a limited impact on the temperature of surrounding environment, i.e., the optical characteristics of VO_2_ are insensitive to the changes in the Fermi energy level of graphene. Consequently, these two tunable methods almost do not interfere with each other. In practice, the CVD-grown graphene layer can be transferred onto the multilayer substrate by a transfer technique using polymethylmethacrylate (PMMA) supporting layers, and is subsequently patterned by photolithography and oxygen plasma etching [49]. A vanadium film can be sputtered onto the lower Topas layer at room temperature and subsequently converted into VO_2_ under O_2_ ambient at 375 °C, and then subsequently patterned by e-beam lithography [50].

## 3. Results and Discussion

### 3.1. Broadband Absorber Used for Switch Applications

**Figure 3:**
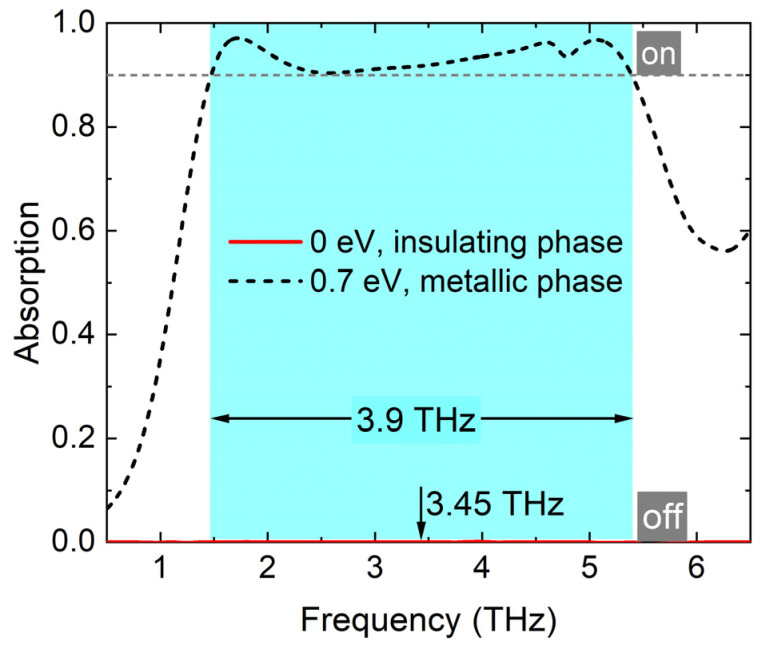
Absorption spectra of the broadband absorber with *E*_f_ = 0 eV, VO_2_ in insulating phase (dashed curve), and *E*_f_ = 0.7 eV, VO_2_ in metallic phase (solid curve).

The intensity of absorptance *A* is expressed by *A* = 1 − *T* − *R*, where transmittance *T* = |*S*_21_|^2^ and reflectance *R* = |*S*_11_|^2^. The absorption spectra of the broadband absorber is shown in Figure 3. The relative bandwidth is 113%. According to the investigation of the active materials, graphene and VO_2_, the switch control can be realized via external voltage and ambient temperature, respectively. When the Fermi energy level of the two graphene layers are set as 0.7 eV, and VO_2_ is in the metallic phase, the proposed absorber is turned ‘on’ from 1.5 to 5.4 THz with the absorptance exceeding 90%, as depicted by the dashed curve. Correspondingly, the proposed absorber turns ‘off’ when the Fermi energy level of the two graphene layers are set as 0 eV, and VO_2_ is in the insulating phase, as depicted by the solid curve. The employment of an electric voltage as the stimuli contributes to a fast switch, while the thermal accumulation takes some time. Thus, the response time of switching from the ‘off’ to the ’on’ state takes from picoseconds to several minutes depending on the strength and duration of excitation, as well as the initial temperature and thermal mass of the VO_2_ component [51,52]. Modulation depth (MD) is introduced to measure the performance for switch application, which can be expressed by *MD* = (*A*_max_ − *A*_min_)/*A*_max_. Then, a THz switch with the excellent value of *MD* (97.1%) is realized.

**Figure 4:**
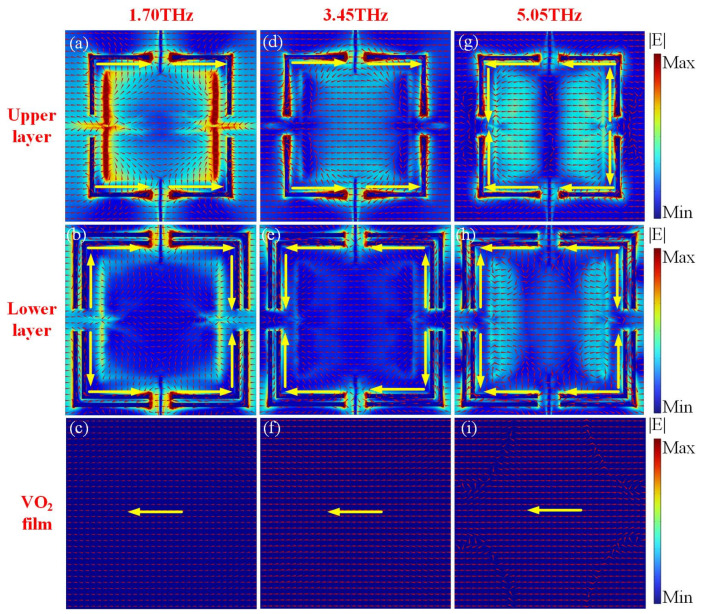
The electric field amplitude |E| distributions for (**a**,**d**,**g**) the lower metamaterial layer, (**b**,**e**,**h**) the upper metamaterial layer, and (**c**,**f**,**i**) the VO_2_ film at 1.70, 3.45 and 5.05 THz, respectively. The normalized surface currents are marked with red short arrows. The yellow long arrows represent the direction of the surface currents on the VO_2_ split loops.

**Figure 5:**
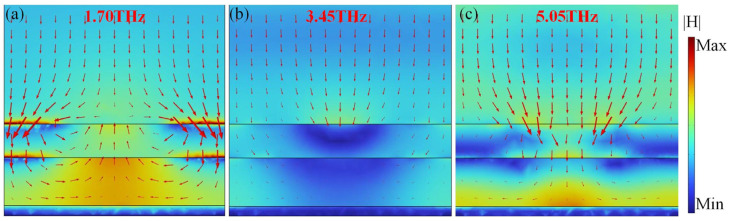
Distributions of the magnetic field |H| and power flow (red arrows) at the central cross section of unit cell (**a**) at 1.70 THz, (**b**) at 3.45 THz, and (**c**) 5.05 THz, respectively.

To clarify the physical mechanism of the proposed absorber, the electric field and the current distributions are investigated. Figure 4a–c show the distributions of the electric field and current at the first resonance, 1.7 THz, for the upper metamaterial layer, the lower metamaterial layer and the VO_2_ film, respectively. The electric field is concentrated between the top and bottom gaps of VO_2_ split loops, and also on the edges. The direction of surface currents on the graphene layers is parallel to the electric field of the incident wave, which indicates an electric resonance [53]. The direction of the currents (represented by the yellow long arrows) on the horizontal VO_2_ splits of metamaterial layers (both the upper and lower layers) are antiparallel to those of the currents on the VO_2_ film, which form a loop and result in a strong magnetic resonance, as shown in Figure 4a,b. In contrast, the currents on the vertical VO_2_ splits are negligible. The investigation can be confirmed by the distributions of the magnetic field |H| in Figure 5a. For the vertical VO_2_ splits in the lower metamaterial layer, the currents show an opposite direction, which can be viewed as an electric quadrupole moment [54]. In addition, for the horizontal VO_2_ splits in both the upper and lower metamaterial layers, the currents show the same direction, which can be viewed as an electric dipole moment.

The current distributions of the upper metamaterial layer and the VO_2_ film at the center frequency 3.45 THz are similar to that at 1.7 THz, as shown in Figure 4d,f. The currents on horizontal VO_2_ splits in lower metamaterial layers are all heading in the same direction, while the currents on the vertical splits show opposite directions. Therefore, this phenomenon results in the electric dipole moment and electric quadrupole moment. The distributions of the magnetic field |H| at 3.45 THz are shown in Figure 5b. The electric field amplitude distributions at the second resonance, 5.05 THz, show electric dipole moment and electric quadrupole moment, as indicated in Figure 4g,h. Different from the resonance frequency, 1.7 THz, Figure 4h,i show that the magnetic resonance at 5.05 THz can be attributed to the antiparallel currents between the lower graphene layer and the VO_2_ film, and then a strong magnetic field is obtained, as shown in Figure 5c. Therefore, profiting from the combination of magnetic resonance and electrical resonance, the incident waves are consumed, and an excellent broadband absorption can be achieved from 1.5 to 5.4 THz.

**Figure 6:**
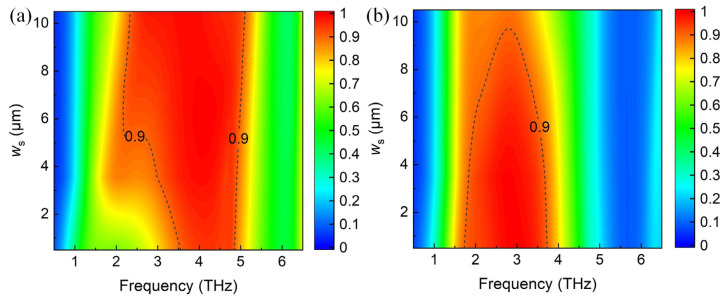
The absorption spectra varying with *w*_s_ (**a**) without the lower metamaterial layer, and (**b**) without the upper metamaterial layer.

In order to further distinguish the influences of the upper and lower metamaterial layers, we investigate the absorption spectra varying with *w*_s_ without the upper or lower layer, as shown in Figure 6. It is clear that the proposed absorber with a single metamaterial layer will result in a reduction in bandwidth. The upper metamaterial layer mainly affects the high frequency absorption, as shown in Figure 6a, while the lower metamaterial layer plays an important role in the low frequency absorption, as shown in Figure 6b. Combining the influence of *w*_s_ on the absorption spectra in Figure 6a,b, *w*_s_ is optimized as 5.5 μm in the simulation.

**Figure 7:**
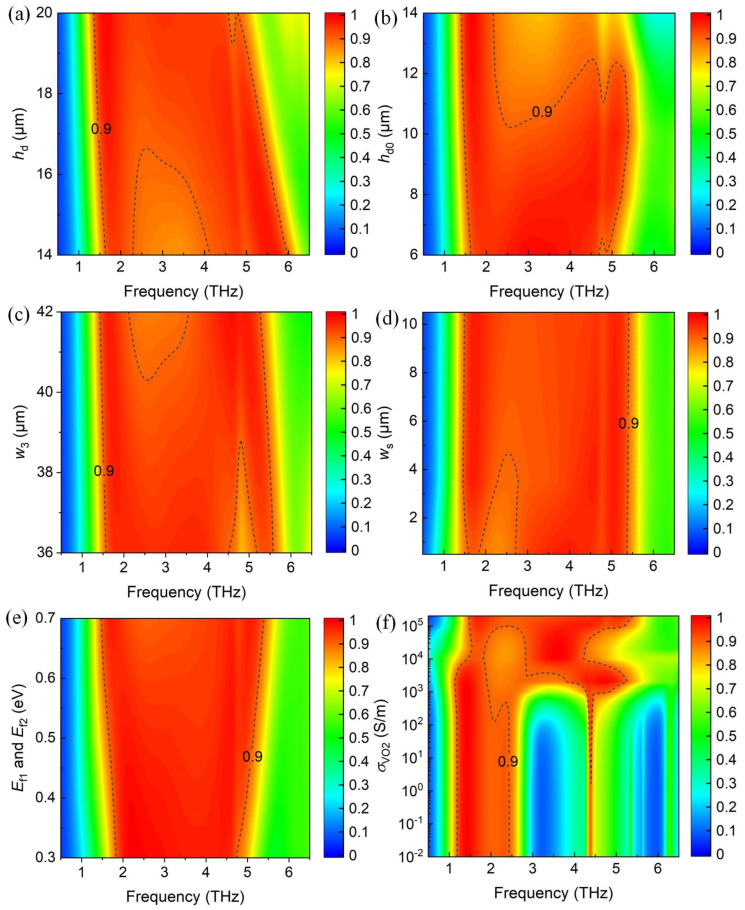
The simulated absorption spectra of the broadband absorber as a function of (**a**) the space *h*_d_ between the upper metamaterial layer and VO_2_ film, (**b**) the space *h*_d0_ of the two metamaterial layers, (**c**) the width of VO_2_ split *w*_3_, (**d**) the gap of VO_2_ split *w*_s_. The absorption spectra vary with (**e**) *E*_f_ varying from 0.3 to 0.7 eV, and (**f**) the conductivity of VO_2_ from 10^−2^ to 2 × 10^5^ S/m.

Subsequently, the influence of key structural parameters on the broadband absorption is investigated to guide the real fabrication of the proposed absorber, as shown in Figure 7. It can be seen from Figure 7a that when *h*_d_ increases, the first and second resonances appear red-shifted with different degrees due to the influence of the magnetic resonance, and the absorption bandwidth narrows. Figure 7b shows the influence of the space *h*_d0_ between the two metamaterial layers with a fixed value of *h*_d_. It is clear that the first resonance varies slightly, while the second resonance shows significant changes. This phenomenon can be explained by the *h*_d1_ decrease as the increment of *h*_d0_ for the fixed *h*_d_. Then, the decreasing *h*_d1_ influences the magnetic resonance between the lower graphene layer and the VO_2_ film. In Figure 7c, the condition is set as: when *w*_3_ increases, *w*_1_ and *w*_2_ also increase, with a fixed spacer and width of VO_2_ loops. Thus, the two resonances show red shift due to the increasing effective length of the LC circuit model. The variety of *w*_s_ has little influence on the absorption bandwidth, as shown in Figure 7d. The influence of Fermi energy level and the conductivity of VO_2_ on absorption spectra is investigated in Figure 7e,f, respectively. In order to optimize the parameters, 0.7 eV and 2 × 10^5^ S/m is chosen as the final value of Fermi energy level and the conductivity of VO_2_, respectively.

### 3.2. Multiple Broadband Absorption Modes

**Figure 8:**
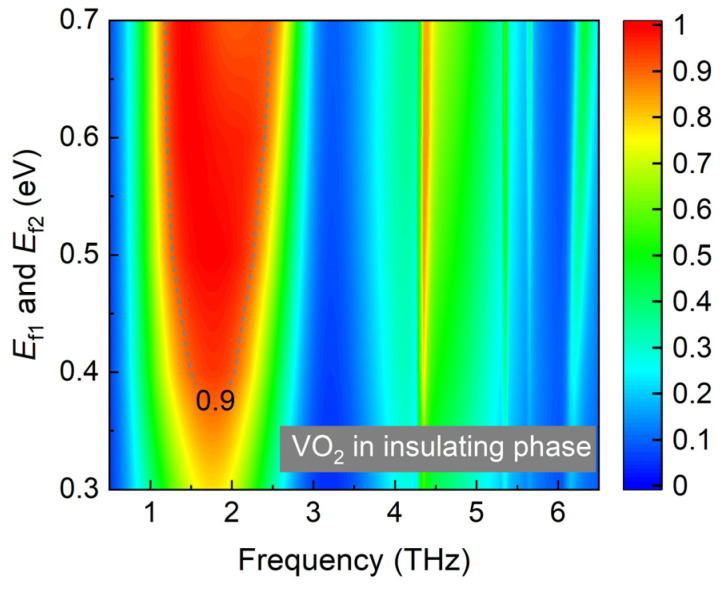
The simulated absorption spectra of the proposed structure with VO_2_ in insulating phase, and the Fermi energy level varies from 0.3 to 0.7 eV.

**Figure 9:**
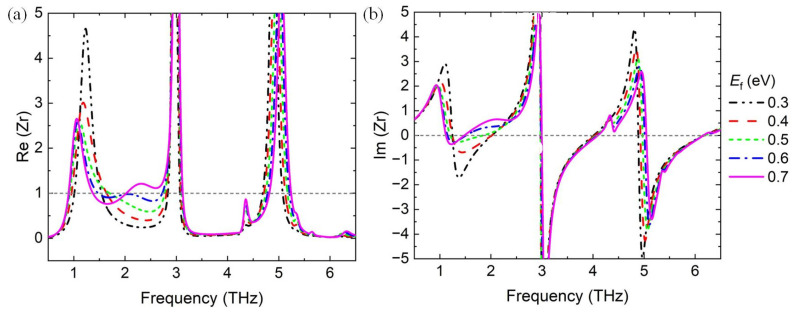
(**a**) Real part and (**b**) imaginary part of the relative impedance Zr with different Fermi energy levels of graphene.

The proposed broadband absorber can further realize multiple broadband absorption modes, utilizing the active materials graphene and VO_2_. Figure 8 shows the absorption spectra of the proposed absorber in the insulating phase, and the Fermi energy level varies from 0.3 to 0.7 eV. The bandwidth of the absorption spectra ranges from 1.2 to 2.45 THz with *E*_f1_ = *E*_f2_ = 0.7 eV. The amplitude can also be adjusted by tuning the Fermi energy level, which can be well elucidated by the impedance-matching theory, as shown in Figure 9a,b.

The amplitude of the absorption coefficient depends on the matching degree between the equivalent value of the proposed absorber and the impedance of free space. The transmittance is nearly zero, as the bottom gold plane prevents the propagation of the incident wave. Thus, the absorption coefficient *A* can be expressed as follows:

(4) $$A\left(\omega \right)=1-R\left(\omega \right)=1-\left|\frac{Z_{r}-1}{Z_{r}+1}\right|,$$

(5) $$Z_{r}=\frac{z_{1}}{z_{0}}=\sqrt{\frac{{\left(1+S_{11}\left(\omega \right)\right)}^{2}-S_{21}{\left(\omega \right)}^{2}}{{\left(1-S_{11}\left(\omega \right)\right)}^{2}-S_{21}{\left(\omega \right)}^{2}}},$$

where *Z*_0_ is the impedance of free space, and *Z*_1_ is the equivalent impedance of the proposed absorber. When the relative impedance *Z*_r_ = 1, the absorption performance is perfect. Figure 9a,b show the real part and imaginary part of the relative impedance *Z*_r_ with different Fermi energy levels, respectively. The results shows that Re(*Z*_r_) is close to 1, and Im(*Z*_r_) approaches zero in the frequency range from 1.2 to 2.45 THz for *E*_f_ = 0.7 eV, indicating excellent absorption performance. The results are consistent with the analysis in Figure 8.

**Figure 10:**
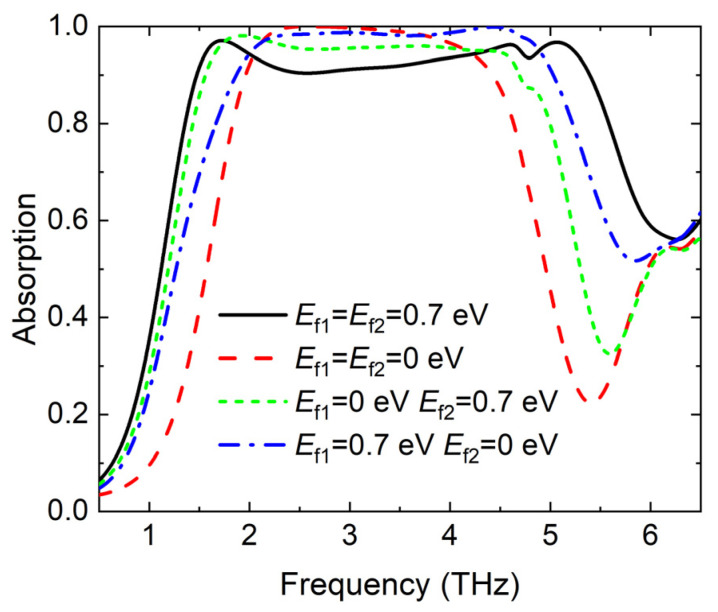
The simulated absorption spectra of the proposed absorber with different Fermi energy levels of the upper and lower graphene layers.

Furthermore, the absorption spectra of the proposed absorber are investigated with different Fermi energy levels of the graphene layers, as illustrated in Figure 10. The other three broadband absorption modes can be achieved with VO_2_ in the metallic phase. In general, the proposed absorber can realize a variable absorption bandwidth through the active materials graphene and VO_2_.

## 4. Conclusions

In conclusion, a metamaterials-based terahertz absorber with the active materials graphene and VO_2_ is designed for broadband switch applications with multiple broadband absorption modes. The dual metamaterial layers of the proposed absorber consist of periodical arrays of graphene squares with VO_2_ split loops. The broadband absorption is more than 90% from 1.5 to 5.4 THz, and the relative bandwidth is 113%. By electrically and thermally controlling the Fermi energy level of graphene and the conductivity of VO_2_, the multiple broadband absorption modes can be realized. When the Fermi energy levels of graphene are both set as 0 eV, and VO_2_ is in the insulating phase, the proposed absorber turns ‘off’. Moreover, the physical mechanism of the absorption properties is also analyzed, and the result reveals that the broadband absorption benefits from the combination of magnetic resonance and electrical resonance. Compared with other reported functional absorbers, the proposed absorber, with a larger relative bandwidth and dynamically variable properties, can be used for terahertz switch, which shows potential applications in terahertz cloaking, switching, and so on.

## Figures and Tables

**Table 1:** The comparison between our work and other multifunctional absorbers.

Reference	Functionality	Switchable States	RB of Broadband	Active Materials	Tunning Method
[39]	Single- and dual-band absorption	2	\	SrTiO_3_ and Dirac semimetal	Temperature and voltage
[40]	Broadband and narrowband	2	111%	Graphene and VO_2_	Temperature and voltage
[41]	Broadband and narrowband	2	97.1%	Graphene and VO_2_	Temperature and voltage
[42]	Single narrowband	1	\	Graphene, SrTiO_3_ and VO_2_	Temperature and voltage
[43]	Triple-band and broadband	2	82.5%	VO_2_	Temperature
[44]	Multiband (three peaks) and broadband	2	122.6%	Graphene and VO_2_	Temperature and voltage
[45]	Broadband and narrowband	2	66.7%	Graphene	Voltage
This study	Broadband absorption with variable bandwidth	5	113%	Graphene and VO_2_	Temperature and voltage

**Table 2:** Detailed dimensions of the proposed absorber.

Parameter	Description	Numerical Value
*P_x_*	Period of unit cell in the *x*-direction	46 μm
*P_y_*	Period of unit cell in the *y*-direction	46 μm
*h_d_*_0_	Spacer of the two graphene layers	7 μm
*h_d_*_1_	Thickness of the upper Topas layer	10 μm
*h_d_*_2_	Thickness of the lower Topas layer	8 μm
*h_VO_*_2_	Thickness of the VO_2_ layer	2 μm
*w_s_*	Gap of the VO_2_ split loops	5.5 μm
*w*_1_	Length of the VO_2_ split loops	42 μm
*w*_2_		38 μm
*w*_3_		34 μm
*s*_1_	Length of the graphene squares	28 μm
*s*_2_		26 μm

## Data Availability

The original contributions presented in the study are included in the article, further inquiries can be directed to the corresponding authors.
